# 141. Evaluating Cefepime And Alternative Beta-lactams For The Treatment Of Serratia marcescens Blood Stream Infections

**DOI:** 10.1093/ofid/ofac492.219

**Published:** 2022-12-15

**Authors:** Natcha Vicente-Lopez, Radha Patel, Kent J Stock

**Affiliations:** Roper, charleston, South Carolina; Roper, charleston, South Carolina; Low Country Infectious Disease, Charleston, South Carolina

## Abstract

**Background:**

AmpC β-lactamase enzymes can be produced by a number of Enterobacterales. Due to its inducible chromosomal resistance, cefepime is often preferred. In vitro analysis and clinical reports have shown AmpC expression can occur less than 5% among S. marcescens. Locally, there has been a rise in beta-lactam resistant S. marcescens, however guidelines and small clinical trials have suggested treatment according to susceptibility testing. This study aimed to evaluate the use of cefepime vs. alternative beta-lactams like third generation cephalosporins or piperacillin-tazobactam as treatment of S. marcescens.

Overall treatment Failure

**Figure:**
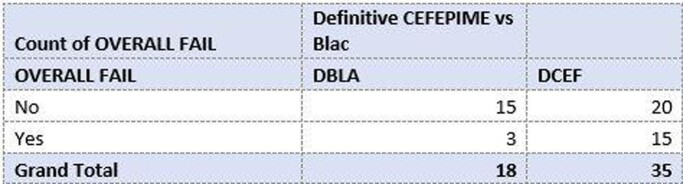

**Methods:**

This is a single-center, retrospective review of adult hospitalized patients with S. marcescens BSIs over a five-year period. Patients who received more than 24 hours of susceptible antibiotics were included. Patients who received at least 72 hours of antibiotics from index blood culture were divided into definitive cefepime (DCEF) or definitive alternative beta-lactams (DBLA) groups. Composite outcome of 30-day re-admission, 90-day reinfection rates, and mortality was used to evaluate treatment failure.

**Results:**

A total of 53 patients were enrolled. Common sources of infection include genitourinary (12), bone and joint (6), and skin and soft tissue (6). DCEF and DBLA groups included 35 and 18 patients, respectively. Most DBLA patients received piperacillin-tazobactam (9/18). Median Charlson Comorbidity Index (CCI) was 6 for DCEF and 4.5 for DBLA. 11 patients in the DCEF group were readmitted, vs 2 patients in the DBLA group. One patient in each group had reinfection within 90 days. In-hospital mortality occurred in 4 and 1 patients in the DCEF and DBLA groups, respectively. Overall treatment failure was observed in 15 patients in the DCEF group and 3 patients in the DBLA group. Median hospital length of stay was 10.1 days for DCEF and 9.5 days for DBLA.

**Conclusion:**

More patients received DCEF compared to DBLA, potentially related to acuity evidenced by higher CCI and ICU admissions. Results showed a higher rate of overall treatment failure among DCEF group. Due to the retrospective design and small sample size, it is difficult to infer clinical significance. These findings prompt further investigation into the difference in treatment failure between these groups.

**Disclosures:**

**All Authors**: No reported disclosures.

